# The recombinant expression and activity detection of MAF-1 fusion protein

**DOI:** 10.1038/srep14716

**Published:** 2015-10-01

**Authors:** Ping Fu, Jianwei Wu, Song Gao, Guo Guo, Yong Zhang, Jian Liu

**Affiliations:** 1 Department of Parasitology, Pathogen Biology Laboratory, Guizhou Medical University, No 9 Beijing Road, Guiyang, Guizhou Province, 550004, P.R.China; 2 Department of Internal Medicine, Center for Cardiovascular Biology and Atherosclerosis Research, The University of Texas Medical School at Houston, 6431 Fannin Street, MSB 1.246; Houston, Texas, 77030, USA

## Abstract

This study establishes the recombinant expression system of MAF-1 (*Musca domestica* antifungal peptide-1) and demonstrates the antifungal activity of the expression product and shows the relationship between biological activity and structure. The gene segments on mature peptide part of MAF-1 were cloned, based on the primers designed according to the cDNA sequence of MAF-1. We constructed the recombinant prokaryotic expression plasmid using prokaryotic expression vector (pET-28a(+)) and converted it to the competent cell of BL21(DE3) to gain recombinant MAF-1 fusion protein with His tag sequence through purifying affinity chromatographic column of Ni-NTA. To conduct the Western Blotting test, recombinant MAF-1 fusion protein was used to produce the polyclonal antibody of rat. The antifungal activity of the expression product was detected using *Candida albicans* (ATCC10231) as the indicator. The MAF-1 recombinant fusion protein was purified to exhibit obvious antifungal activity, which lays the foundation for the further study of MAF-1 biological activity, the relationship between structure and function, as well as control of gene expression.

*Musca domestica* antifungal peptide −1 (MAF-1) was a novel insect antifungal peptide obtained from the 3rd instar of *Musca domestica* larvae. The cDNA sequence has been determined and a full-scale bioinformatics analysis was provided^1^,^2^. In order to further establish the MAF-1 recombinant expression system and obtain active recombinant protein, the cDNA sequence of the clone of MAF-1 and the bioinformatics analysis results need to be verified. This can lay the foundation for the further study of MAF-1 biological activity, the relationship between structure and function and metabolic kinetics. It also will be helpful for producing artificial MAF-1 protein. However the low yield obtainable from *in*-*vivo* immune induction of insect antimicrobial peptides, procedures for extracting it from the organisms and exorbitant price of direct synthesis, all severely limited the developments and applications of the peptides^3^,^4^,^5^,^6^. Genetic engineering expression is therefore the preferred approach for producing antimicrobial peptides. However antimicrobial peptide molecules are very small and will most likely be decomposed by protease. Meanwhile, because the expression product is harmful to the host bacteria, it usually can’t be expressed in a pronucleus system and therefore eukaryotic or fusion expressions are generally employed. In recent years, successful fusion expressions of various antimicrobial peptides in prokaryotic cell have been reported^7^,^8^,^9^,^10^,^11^. The *escherichia coli* expression system with lucid genotypic milieu, high level gene expression, short cultivation period and easily purified tag protein, was the earliest and preferred expression system applied to antimicrobial peptides and heterologous proteins^12^. The authors have applied it to the fusion expression of MAF-1 gene and His tag. Using this procedure the expression product, namely recombinant MAF-1 protein, carries a His tag, which reinforces stability and expedites purification of the recombinant protein^13^,^14^,^15^,^16^,^17^.

## Results

### Constructed MAF-1 gene expression vector

**Figure 1:**
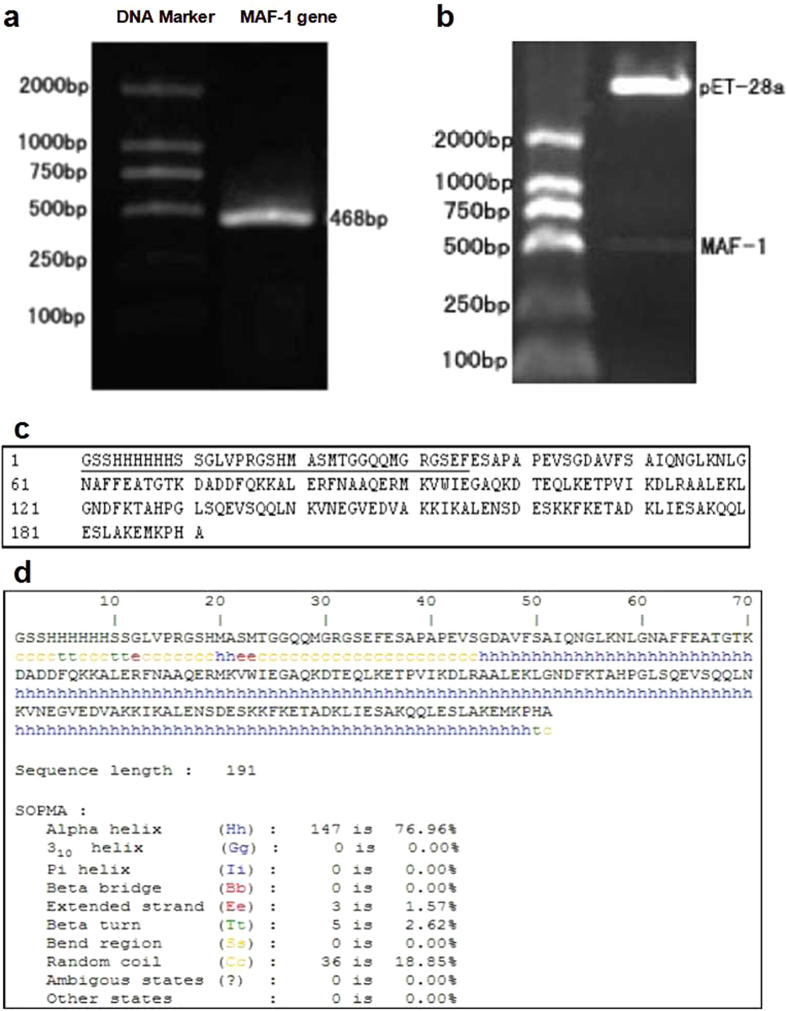
Constructed MAF-1 gene expression vector and analyzed the fusion protein. (**a**) The result of PCR for MAF-1. (Lane 1, DL2000 DNA Marker. Lane 2, MAF-1 gene). (**b**) Electrophoresis of digesting recombinant plasmid pET-28a(+)-MAF-1. (Lane 1, DL2000 DNA Marker. Lane 2, EcoRI and Hind III digestion of recombinant plasmid pET-28a(+)-MAF-1). (**c**) The amino acids sequence of MAF-1 fusion protein. (___, The insert amino acid sequence). d:The secondary structure prediction for MAF-1 fusion protein.

Taking the 3rd instars of *Musca domestica* larvae RNA as a template and amplifying the mature peptide fragment of MAF-1 (468 bp) through PCR, a distinctive stripe near visible 500 bp was identified (Fig. 1a) when 1% agarose gel electrophoresis was performed. The digestion products from the double digestion with *Eco*R I and *Hin*d III to pET-28a(+)-MAF-1 recombinant plasmid was analyzed with 1% AGE. It was shown that there is a clear stripe visible near 500 bp (Fig. 1b), which was about the size of target gene. In addition, the sequencing report of the recombinant plasmid insertion sequence agrees with theoretical sequence, which shows successful establishment of recombinant plasmid. Analyzed with DNAman software it was shown that the recombinant MAF-1 protein sequence was a 156 N-terminals amino acid sequence of the MAF-1 mature peptide with an added 35 amino acid residue, which contained the His label sequence (Fig. 1c). The secondary structure of recombinant MAF-1 fusion protein, analysed with SOPMA tools of ExPASy, demonstrated that the introduction of the His label retained the secondary structural nature of the MAF-1 mature peptide (Fig. 1d).

### MAF-1 recombinant protein expression

**Figure 2:**
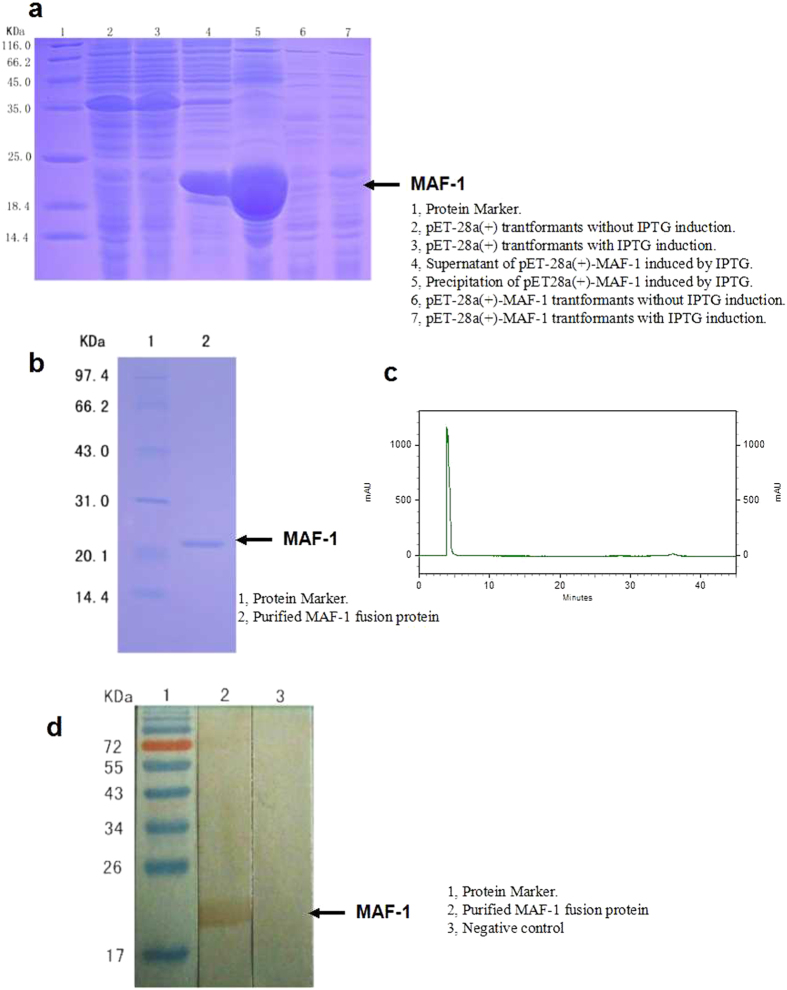
Identification of recombinant MAF-1 fusion protein. (**a**) SDS-PAGE analysis of MAF-1 fusion protein. (Lane 1, Protein Marker. Lane 2, pET-28a(+) trantformants without IPTG induction; Lane 3, pET-28a(+) trantformants with IPTG induction. Lane 4, Supernatant of pET-28a(+)-MAF-1 induced by IPTG. Lane 5, Precipitation of pET28a(+)-MAF-1 induced by IPTG. Lane 6, pET-28a(+)-MAF-1 trantformants without IPTG induction. Lane 7, pET-28a(+)-MAF-1 trantformants with IPTG induction). (**b**) SDS-PAGE analysis of the purified MAF-1 fusion protein. (Lane 1, Protein Marker. Lane 2, Purified fusion protein of pET28a(+)-MAF-1). (**c**) RP-HPLC peaks of MAF-1 fusion protein. (**d**) Western Blotting result of MAF-1 fusion protein. (Lane 1, Protein Marker. Lane 2, Fusion protein of pET28a(+)-MAF-1. Lane 3, Negative control).

The product of the pET-28a(+) – MAF-1 recombinant plasmid in *Escherichia coli* (BL21/DE, through induction at 37 °C 1 mmol/L IPTG, with 12% SDS-PAGE) was compared to pET-28a(+) no-load transformed bacteria and the induction group of recombinant plasmid transformed bacteria without IPTG. A clear expression stripe of protein was found near the molecular weight standard between 18.4 kD and 25.0 kD (the sum of MAF-1 mature peptide and His-tag molecular weight). Moreover, the supernatant of recombinant plasmid transformed bacteria after splitting contained soluble recombinant MAF-1 fusion protein and a lot of recombinant MAF-1 fusion protein expressions were found in the inclusion body (Fig. 2a).

Using a His Bind Purification Kit a purified recombinant MAF-1 fusion protein (from body and bacteria fluid supernatant of pET-28a (+)-MAF-1 recombinant plasmid transformed bacteria) could be completely eluted by an elution buffer solution containing 50 mM 100 mM imidazole (Fig. 2b). After concentration and dialyzation of the eluent the concentration of albumen was measured as 0.401 mg/mL. As shown in the RP-HPLC image, a major absorbed peak was observed at 4 minutes (Fig. 2c).

Western Blot identification of purified recombinant MAF-1 fusion protein showed that recombinant MAF-1 fusion protein could be recognized by prepared rat anti recombinant MAF-1 fusion protein immune serum. The molecular weight of developing band appeared in the expected position (Fig. 2d).

### MAF-1 fusion protein anti-fungal activity detection

**Figure 3:**
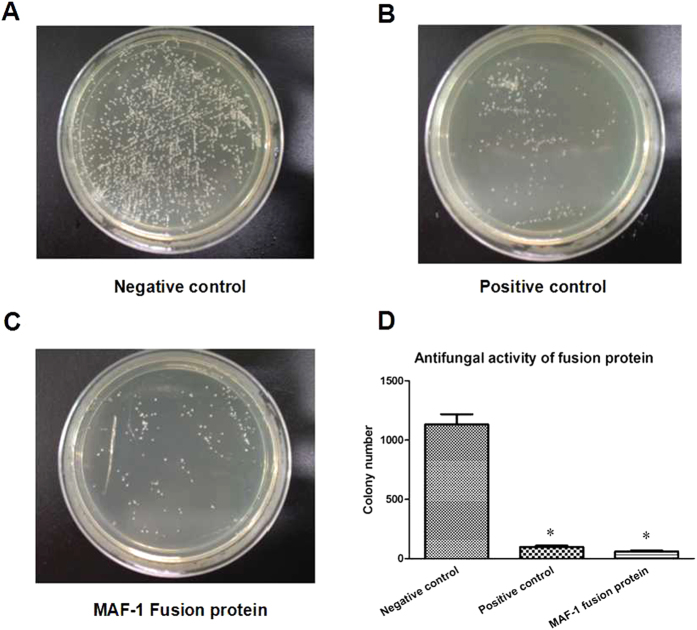
Antifungal activity detection of recombinant MAF-1 fusion protein. (**a**) Negative control. (**b**) Positive control. (**c**) MAF-1 fusion protein. (**d**) Antifungal activity of fusion protein. Data represent means ± SD. *P < 0.05 Compared with Negative control.

Taking *candida* albicans (ATCC10231) as the indicator, the antifungal activity of recombinant MAF-1 fusion protein after purification was detected by Micro liquid–bacterial colony notation. The images shown that 100 μg/mL recombinant MAF-1 had the obvious antifungal activity. Compared with negative control group, the colony number of MAF-1 treatment group was significant decreased (Fig. 3).

## Discussion

Antimicrobial peptides are the key components of the insect immune system. It is also an insect’s first defense line in resisting invasion by pathogenic microorganisms. Anti-microbial peptides are widely distributed among different insects and the presence of these functional peptides varies from species to species^18^,^19^,^20^,^21^,^22^,^23^. These peptides are heat-resistant and advantageously cause no damage to the cells of higher animals and hence these peptides have been regarded as new sources for antibacterial and antifungal agents. The antimicrobial peptides of musca are being extensively studied, but research reports on the isolation, purification, expression and cloning of antimicrobial peptides are rather scarce.

Genetic engineering has now become the most frequently used method of obtaining antimicrobial peptides due to the low content of antimicrobial peptides in insects and the difficulty and high cost of purification. Yuan *et al.* conducted research transferring Drosomycin gene to *Escherichia coli*, the result showing that the expression products possess antifungal effect^24^. Analyzing the Heliomicin gene, Lamberty *et al.* determined the sequence structure of the defensin insect family. The expression products from recombinant expression inside yeast maintained resistance to bacteria and fungal infection^25^. Kim *et al.* built an *E.coli* expression system of tenecin III, which makes mass production of recombinant tenecin III with anti *C.albicans* activity possible^26^. The prokaryotic expression of insect antifungal peptide gene and expressions in yeast has also been reported, the expression products all maintaining resistance to fungal growth^27^,^28^,^29^.

The expression system of *escherichia coli* has the advantages of clear genotypic background, high level of target gene expression, short cultivation cycle and carrying easily purified tag proteins. It is the earliest expression system applied to express antimicrobial peptide. Using prokaryotic expression system to heterologously express antimicrobial peptides is now mature, however fusion proteins are usually used to enhance stability of antimicrobial peptides^30^. pET carrier series are a group of expression carriers used for protein or polypeptide high level expression and purification. The pET-28a (+) selected in this study can make heterologous gene and His tag fuse expression, which enables facile recombinant fusion protein purification and enhanced recombinant protein stability and immunogenicity.

In the process of purifying recombinant MAF-1 fusion protein (using the His·Bind Purification Kit) from the bacteria fluid supernatant of pET-28a(+)-MAF-1 recombinant plasmid bacteria the recombinant protein did not remain on the column. At present, the usual reason that recombinant protein with added His tag does not remain on the column is that His tags do not ionize and can’t be folded inside the protein or adhere to the protein itself. No chemical bond can be formed, the protein has no affinity with Ni, and cannot adsorb onto the column. Denaturant urea may break covalent bonds, ionic bonds, induce a hydrophobic effect, or affect the electrostatic interaction within or between protein molecule and make polypeptide chains extend^31^. In this study urea added to the bacteria fluid (to a final concentration of 1 M) allowed the recombinant fusion protein to adsorb onto the column. Collecting eluant to dialyze can not only eliminate the use of urea and re-nature proteins, but can also remove Ni ion from sample.

Exogenous proteins of high expression in *E.coli* cytoplasm, especially those from eucaryon, usually form insoluble polymers named inclusion bodies^32^. When expressions are recombined, inclusion bodies containing recombinant MAF-1 fusion protein are formed in bacteria fluid of transformed bacteria. In general, inclusion bodies are high in protein expression, but purification requires degeneration and renaturation. After urea treatment methods of protein renaturation were employed before column purification. Dialysis is used in the process of renaturation and the urea concentration in the sample can be decreased. Urea was also added to solute recombinant proteins in the supernatant bacterial fluid before column purification. The final urea concentration reaches 1 M during renaturation. In this way solute recombinant protein in the bacteria fluid of transformed bacteria and in recombinant protein in inclusion body was purified and recycled to increase yield of recombinant protein.

The result of the antifungal activity test shows that although the His tag of recombinant MAF-1 fusion protein N-terminal hasn’t been removed, it is still provided with obvious antifungal activity at a concentration of 100 μg/mL. This is also true for mature MAF-1 when the concentration is at 72.92 μg/mL^1^. However further experimentation is needed in the comparison of antifungal activity between recombinant MAF-1 and mature MAF-1. This will involve the study of the antibacterial spectrum, minimum bactericidal concentration (MBC) and minimum inhibitory concentration (MIC) of recombinant MAF-1 fusion protein, and the determination of various factors influencing the antifungal activity. In this study, the expression and purification method of MAF-1 has been successfully established in prokaryotic cell and the recombinant MAF-1 fusion protein with antifungal activity has been obtained. This lays the foundation for further study on the biological activity of MAF-1, the relationship between structure and function, as well as control of gene expression.

## Materials and Methods

### Materials

The use of all material for this study was approved by the institutional review board of Guiyang Medical University, China. All following methods/experimental protocols were carried out in accordance with the approved guidelines of the Ethics committee of Guiyang Medical University, China.

PrimeScript™ One Step RT-PCR Kit, Ex Taq enzyme, *EcoR I* and *Hind III* restriction endonucleases, T4DNA ligase, dNTP Mixture, Primer STARTM HS DNA Polymerase, DNA Label DL2000 and 1 Kb DNA ladder were all purchased from TakaRa Company in Da Lian. A His Band Purification Kit was purchased from Novagen Company. Complete Freund’s adjuvant and incomplete Freund’s adjuvant were purchased from Sigma Company. *Escherichia coli* DH5α, BL21/DE3 and plasmid pET-28a(+) were obtained from Sun Yat-sen University and stored at the Pathogeny Biology Laboratory of GuiYang Medical University. *Candida albicans*(ATCC10231) was stored at our laboratory. The other reagents were available in our laboratory and were analytically pure.

*Musca domesticals* was laboratory bred. SD(Sprague Dawley), male, aged from 6 to 8 weeks, were purchased from the laboratory animal centre of GuiYang Medical University.

The primer was compounded by TakaRa Company in Da Lian. The forward primer is 5′GGAATTCGAATCTGCCCCCGCCCCT GAGGT-3′ and the reverse primer is 5′- CCCAAGCTTCTAGGCATGGGGCTTCATTTCCTTGGC -3′.

## Methods

### Building the clone and expression vector of MAF-1 gene

The 3rd instar of Musca *domestica* larvae RNA was extracted and used as template for gene amplification. The PCR product was identified by AGE (agarose gel electrophoresis) and then recycled. Plasmid pET-28a(+) was extracted according to the manual of the plasmid extraction kit. Purified MAF-1 gene and plasmid pET-28a(+) were subjected to a *Eco*RI and *Hind*III double digestion separately. T4 NDA enzyme was connected and then converted to *escherichia coli* DH5α. The Recon was screened and sequence comparative analysis was completed. In order to obtain the engineering bacteria (BL21(DE3)/pET-28a(+)-MAF-1) that expresses the MAF-1 fusion protein, the expression plasmid of the correctly sequenced result was converted to the competent cell of *escherichia coli*, BL21/DE.

### Recombinating inducible expression of MAF-1 fusion protein in escherichia coli BL21/DE

A single colony of recombinant expression vector, BL21(DE3)/pET-28a(+)-MAF-1 and control cell single colony containing an empty vector were inoculated in fluid mediums containing 100 μg/mL kanamycin. It was shaken at 250 rpm for 8 hours at 37 °C and transferred at a ratio of 1:100. The transfer liquid was shaken at 37 °C and 250 rpm for cultivation until the OD_600_ was 0.6. The IPTG was added to make the final concentration of solution 1 mmol/L, shaken at 37 °C and 250 rpm for 6 hours. Thalluses before and after induction were collected for a SDS-PAGE test and analysis.

### Recombinating purification of MAF-1 fusion protein

Bacterial liquid collected from the inducible expression were used to collect thallus (4 °C, centrifuged at 8000 rpm for 20 min) and then re-suspended in Lysis buffer (50 mmol/L Tris·HCL pH 8.0, 1 mmol/L EDTA, 0.1 mol/L NaCl). The thallus was disrupted by ultrasound (160W, ultrasound for 1 sec at 2 sec intervals, for 3 min in total) and centrifuged (4 °C, 13000 rpm at 50 mM, 100 mM, 150 mM d for 20 min). The supernatant and precipitate collected separately and the bacterial liquid was submitted to a SDS-PAGE analysis. Supernatant and precipitate collected from thallus was purified separately according to the procedure of the fusion protein purification manual of His Tag Kit and was eluted at different imidazole concentration (50 mM, 100 mM, 150 mM) and the fractions collected separately. Then, the recombinant MAF-1 protein were filtered through a nitrocellulose membrane filter of 0.22 μm pore size (Millipore), and the filtrate was analyzed using RP-HPLC on a Sephasyl C_18_ column (Beckman, System Gold, USA, ODS 0.5 μm, 0.46 × 25 cm). Absorbed material was eluted with a linear gradient of 0–80% solution B (0.1% trifluoroacetic acid in acetonitrile) in solution A (0.1% trifluoroacetic acid in Ultrapure water) over 40 min at a flow rate of 0.8 mL/min.

### Preparing the rat anti recombinant MAF-1 fusion protein immune serum and western blotting

Recombinant MAF-1 protein was mixed with the same volume of complete Freund’s adjuvant (a drop of emulsion won’t diffuse after being dropped on water)^33^. The back and rear foot pad of SD rats were disinfected with iodine and injected with the emulsion (200 μg per rat). After an interval of two weeks recombinant MAF-1 protein is mixed with the same volume of incomplete Freund’s adjuvant and the rats were again immunized in the same way. Blood samples were gathered, from which the serum was separated and stored at −20 °C, one week after the last immunization^34^. The recombinant fusion protein after purification was transferred to a PVDF membrane using SDS-PAGE. The western blotting was conducted with rat anti recombinant MAF-1 fusion protein immune serum being the first antibody and labeled Goat Anti-Rat (IgG) marked with horseradish peroxidase (HRP) being the second antibody. Color development was conducted with a DAB color development kit.

### Detection of anti fungal activity of recombinant MAF-1 fusion protein

In order to have the fungus in logarithmic growth phase, *candida albicans*(ATCC10231) was inoculated in sabouraud agar and incubated at 37 °C for 24 h. After that, 3 to 5 *candida albicans* colonies in logarithmic growth phase were suspended in 5 mL of sterile distilled water and the turbidity was adjusted to 0.5 Mc. Farland standard units, equivalent to 1 × 10^6^ ~ 5 × 10^6^ mycetocyte/mL. 50 L of 09 candida albicans fluid in the logarithmic phase was added to each well on a sterile well plate together with 50 L of the sample to be tested. Sterile distilled water was used as a negative control and fluconazole as a positive control. All were fully mixed and put to wet box at 37 °C for 24 h. Bacteria colonies were counted after 1 μL of each sample was taken from each well and cross coated onto the surface of sabouraud agar and again put into an incubator at 37 °C for 24 h.

## Statistical analysis

All values are reported as means ± SD. Differences were evaluated by Student’s *t* test or One-way analysis of variance (ANOVA). A two-tailed *P* < 0.05 was taken to indicate a statistically significant difference.

## Additional Information

**How to cite this article**: Fu, P. *et al.* The recombinant expression and activity detection of MAF-1 fusion protein. *Sci. Rep.* **5**, 14716; doi: 10.1038/srep14716 (2015).

## References

[b1] Fu P., Wu J. W. & Guo G. Purification and Molecular Identification of an Antifungal Peptide from the Hemolymph of *Musca domestica* (housefly). Cellular & Molecular Immunology. 4, 245–251, 10.1038/cmi.2009.33 (2009). 19728925 PMC4002715

[b2] Fu P., Wu J. W. & Guo G. cDNA Cloning and Sequence Analysis of *Musca domestica* Antifungal Peptide-1 (MAF-1). Chinese Journal of Parasitology and Parasitic Diseases. 4, 279–284 (2011). 21972603

[b3] Qu H. *et al.* The expression of correlative genes of antibacterial materials in housefly larvae. Chinese Journal of Bioprocess Engineering. 4, 14–18, 10.3969/j.issn.1672-3678.2007.04.004 (2007).

[b4] Shan X. F. *et al.* Advances in Heterogenous Expression of Antimicrobial Peptides from Aquatic Animals. Chinese Journal of Veterinary Drug. 11, 34–36, 10.3969/j.issn.1002-1280.2011.11.010 (2011).

[b5] Yi H. Y. *et al.* Insect antimicrobial peptides and their applications. Applied Microbiology and Biotechnology. 13, 5807–5822, 10.1007/s00253-014-5792-6 (2014). 24811407 PMC4083081

[b6] Gao S. *et al.* Effect of different inducible agents on antifungal peptides of housefly larvae and their antifungal activity. Acta Entomologica Sinica. 10, 1009–1015, 10.3321/j.issn:0454-6296. 2007. 10.006 (2007).

[b7] Xu Q. Y. & Zhu J. Y. Cloning and expression of defensin gene of *Musca domestica* larve. China Tropical Medicine. 1, 4–6, 10.3969/j.issn.1009-9727.2005.01.002 (2005).

[b8] Yang X. R. *et al.* Clone of full-length diptericin from *Musca domestica* and its bioinformatics analysis. Chinese Journal of Vector Biology and Control 5, 393–396 (2009).

[b9] Geng H. *et al.* Molecular Cloning and Expression of Attacin from Housefly (*Musca domestica*). Acta Genetica Sinica. 12, 1344–1350 (2004). 15633638

[b10] Gao S. *et al.* MicroRNA-133a regulates insulin-like growth factor-1 receptor expression and vascular smooth muscle cell proliferation in murine atherosclerosis. Atherosclerosis. 1, 171–179, 10.1016/j.atherosclerosis.2013.11.029 (2014). 24401233 PMC4334121

[b11] Wang W. & Zheng X. L. The prokaryotic expression of cecropin A gene in *Musca domesdica* and immunological identification of its expressed products. Chinese Journal of Zoonoses. 5, 439–445, 10.3969/j.issn.1002-2694.2009.05.010 (2009).

[b12] Shan X. F. *et al.* Advances in Heterogenous Expression of Antimicrobial Peptides from Aquatic Animals. Chinese Journal of Veterinary Drug. 11, 34–36, 10.3969/j.issn.1002-1280. 2011.11.010 (2011).

[b13] Peng L. *et al.* High level expression of soluble human defensin2 in *Escherichia coli*. Process Biochem. 12, 2199 (2004).

[b14] Rao X. *et al.* Design and expression of peptide antibiotic hPAB beta as tandem multimers in *Escherichia coli*. Peptides. 5, 721 (2005). 15808901 10.1016/j.peptides.2004.12.016

[b15] Han Z. X. *et al.* Expression of Recombinant Gallinacin-9 from Chicken in *E.coli* and Determination of Its Antimicrobial Activity. Chinese Journal of Anamal and Veterinary Sciences. 10, 1426–1431, 10.3321/j.issn:0366-6964.2008.10.022 (2008).

[b16] Xie F. *et al.* Prokaryotic expression and antibacterial activity of antibacterial peptide Ranalexin gene from *Rana catesbiana*. Journal of Zhejiang University (Agriculture and Life Sciences). 1, 27–32, 10.3785/j.issn.1008-9209.2009.01.003 (2009).

[b17] Gao S. *et al.* Glucose regulated protein 78 prompted scavenger receptor A-mediated secretion cytokine of tumor necrosis factor-α by Raw 264.7 cells. Clinical and Experimental Pharmacology and Physiology 36, 940–944 (2009). 19473344 10.1111/j.1440-1681.2009.05177.x

[b18] Eleanor R. H. *et al.* Antimicrobial Defense and Persistent Infection in Insects. Science 5905, 1257–1259, 10.1126/science.1165265 (2008). 19023083

[b19] Rahuma N., Ghenghesh K. S., Ben A. R. & Elamaari A. Carriage by the housefly (*Musca domestica*) of multiple-antibiotic-resistant bacteria that are potentially pathogenic to humans, in hospital and other urban environments in Misurata, Libya. Annals of Tropical Medicine and Parasitology 8, 795–802 (2005). 16297293 10.1179/136485905X65134

[b20] Robert I. L. Evolution of Antimicrobial Peptides: A View from the Cystine Chapel. Antimicrobial Peptides and Innate Immunity 1–27, 10.1007/978-3-0348-0541-4_1 (2013).

[b21] Wang X. H., Zheng Y. & Xu Y. M. A novel peptide binding to cytoplasmic domain of class A scavenger receptor reduces lipid uptake in differentiated THP-1 macrophages. BBA-biomembrane 1, 76–83, 10.1016/j.bbalip.2008.10.011 (2009). 19049904

[b22] Dolashka P., Moshtanska V., Borisova V., Dolashki A., Stevanovic S., Dimanov T. & Voelter W. Antimicrobial proline-rich peptides from the hemolymph of marine snail Rapana venosa. Peptides. 7, 1477–83, 10.1016/j.peptides.2011.05.001 (2011). 21703315

[b23] Dolashka P., Dolashki A., Voelter W., Van Beeumen J. & Stevanovic S. Antimicrobial activity of peptides from the hemolymph of *Helix lucorum* snails. International Journal of Current Microbiology and Applied Sciences. 4, 1061–1071 (2015).

[b24] Yuan Y., Gao B. & Zhu S. Functional expression of a Drosophila antifungal peptide in *Escherichia coli*. Protein Expr Purif 2, 457–462 (2007). 17169573 10.1016/j.pep.2006.10.024

[b25] Lamberty M. *et al.* Solution structures of the antifungal heliomicin and a selected variant with both antibacterial and antifungal activities. Biochemistry 40, 11995–12003 (2001). 11580275 10.1021/bi0103563

[b26] Kim D. H. *et al.* Bacterial expression of tenecin 3, an insect antifungal protein isolated from *Tenebrio molitor,* and its efficient purification. Mol Cells 6, 786–789 (1998). 9895135

[b27] Deng X. J. *et al.* Expression of the Antifungal Peptide Genes, Drs and Drs–IC From *Drosophila melanogaster* in E.coli and the Activity Detecrion of Their Expression Products. Science of Sericulture 2, 264–267, 10.3321/j.issn:0454-6296.2006.05.005 (2006).

[b28] Sang Y. X. *et al.* Secretive Expression of Insect Antifungal Peptide Genes in *Pichia pastoris* and Antifungal Activity Assay for the Expressed Products. Scientia Agricultura Sinica 4, 842–849, 10.3321/j.issn:0578-1752.2007.04.027 (2007).

[b29] Huang Y. D. *et al.* Synthesis and Prokaryotic Expression of Insect Antifungal Peptide Thanatin Gene. Acta Sericologica Sinica 2, 104–108, 10.3969/j.issn.0257-4799.2002.02.006 (2002).

[b30] Feng X. J., Wang J. H. & Shan A. S. Review on Gene Engineering and Transgenic Expression Strategy of Antimicrobial Peptides. China Biotechnology 3, 63–67, 10.3969/j.issn.1671-8135.2006.03.013 (2006).

[b31] Rao H. L. *et al.* The study on renaturalizing condition of rhIL -6 expressed in *E. coli*. Progress in Microbiology and Immunology 3, 27–30, 10.3969/j.issn.1005-5673.2007.03.008 (2007).

[b32] Kuang A. L. *et al.* Inclusin bogy formation causes and treatment methods. Shanghai Journal of Animal Husbandry and Veterinary Medicine 1, 62–63, 10.3969/j.issn.1000-7725. 2009.01. 041 (2009).

[b33] Zhu L. P. & Chen X. Q. Immunology Common Experimental Methods. People’s Medical Publishing, 16–18 (2000).

[b34] Zhu L. P. & Chen X. Q. Immunology Common Experimental Methods. People’s Medical Publishing, 352–355 (2000).

